# Impairment of muscular endothelial cell regeneration in dermatomyositis

**DOI:** 10.3389/fneur.2022.952699

**Published:** 2022-10-18

**Authors:** D. Lemmer, J. Schmidt, K. Kummer, B. Lemmer, A. Wrede, C. Seitz, P. Balcarek, K. Schwarze, G. A. Müller, D. Patschan, S. Patschan

**Affiliations:** ^1^Department of Nephrology and Rheumatology, University Medical Center Göttingen, Göttingen, Germany; ^2^Immanuel Krankenhaus Berlin, Medical Center of Rheumatology Berlin-Buch, Berlin, Germany; ^3^Department of Neurology and Pain Treatment, Immanuel Klinik Rüdersdorf, University Hospital of the Brandenburg Medical School Theodor Fontane, Rüdersdorf bei Berlin, Germany; ^4^Faculty of Health Sciences Brandenburg, Brandenburg Medical School Theodor Fontane, Rüdersdorf bei Berlin, Germany; ^5^Department of Neurology, Neuromuscular Center, University Medical Center Göttingen, Göttingen, Germany; ^6^Department of Physics, Georg-August-University Göttingen, Göttingen, Germany; ^7^Department of Neuropathology, University Medical Center Göttingen, Göttingen, Germany; ^8^Department of Dermatology, Allergology and Venereology, University Medical Center Göttingen, Göttingen, Germany; ^9^Department of Trauma Surgery, Orthopedics and Plastic Surgery, University Medical Center Göttingen, Göttingen, Germany; ^10^Arcus Klinik, Pforzheim, Germany; ^11^Department of Medicine 1, Cardiology, Angiology, and Nephrology, University Hospital Brandenburg of the Brandenburg Medical School Theodor Fontane, Branderburg, Germany

**Keywords:** endothelial (dys) function, myositis - etiology, progenitor cells, angiogenic mediators, regeneration

## Abstract

**Background and aim:**

Inflammatory myopathies are heterogeneous in terms of etiology, (immuno)pathology, and clinical findings. Endothelial cell injury, as it occurs in DM, is a common feature of numerous inflammatory and non-inflammatory vascular diseases. Vascular regeneration is mediated by both local and blood-derived mechanisms, such as the mobilization and activation of so-called proangiogenic cells (PACs) or early endothelial progenitor cells (eEPCs). The current study aimed to evaluate parameters of eEPC integrity in dermatomyositis (DM), compared to necrotizing myopathy (NM) and to non-myopathic controls.

**Methods:**

Blood samples from DM and NM patients were compared to non-myositis controls and analyzed for the following parameters: circulating CD133^+^/VEGFR-2^+^ cells, number of colony-forming unit endothelial cells (CFU-ECs), concentrations of angiopoietin 1, vascular endothelial growth factor (VEGF), and CXCL-16. Muscle biopsies from DM and NM subjects underwent immunofluorescence analysis for CXCR6, nestin, and CD31 (PECAM-1). Finally, myotubes, derived from healthy donors, were stimulated with serum samples from DM and NM patients, subsequently followed by RT-PCR for the following candidates: IL-1β, IL-6, nestin, and CD31.

**Results:**

Seventeen (17) DM patients, 7 NM patients, and 40 non-myositis controls were included. CD133^+^/VEGFR-2^+^ cells did not differ between the groups. Both DM and NM patients showed lower CFU-ECs than controls. In DM, intramuscular CD31 abundances were significantly reduced, which indicated vascular rarefaction. Muscular CXCR6 was elevated in both diseases. Circulating CXCL-16 was higher in DM and NM in contrast, compared to controls. Serum from patients with DM but not NM induced a profound upregulation of mRNS expression of CD31 and IL-6 in cultured myotubes.

**Conclusion:**

Our study demonstrates the loss of intramuscular microvessels in DM, accompanied by endothelial activation in DM and NM. Vascular regeneration was impaired in DM and NM. The findings suggest a role for inflammation-associated vascular damage in the pathogenesis of DM.

## Introduction

Inflammatory myopathies (myositis) belong to a heterogeneous group of diseases in terms of etiology, (immuno)pathology, and clinical findings. Clinically, dermatomyositis (DM), immune-mediated necrotizing myopathy (NM), and polymyositis (PM) may present with either acute or subacute / chronic onset and typically become manifest in proximal muscle weakness of variable degree (1). These rare diseases may be associated with extra-muscular manifestations, such as the involvement of lungs, skin, or heart (2). Furthermore, myositis-specific and myositis-associated antibodies play an increasingly important role in terms of clinical phenotypes (2, 3).

DM is considered to be mediated by the complement system, including C5b-9-mediated endothelial injury. Immunoglobulin deposition (4) and perimysial / perivascular CD4^+^ T-cell and B-cell infiltration are typical hallmarks (5).

NM presents histopathological features of muscle necrosis and muscle regeneration with almost no signs of inflammation (6). Positive immunostaining for complement C5b-9 on endomysial capillaries in some cases reveals an associated microvasculopathy comparable to DM (5).

In this study, patients with DM and NM were selected because of similar, yet distinct pathomechanisms.

Endothelial cell injury, as it occurs in DM, is a common feature of numerous inflammatory and non-inflammatory vascular diseases, such as atherosclerosis, vasculitis, sepsis, and thrombotic microangiopathy (7–10). In recent years, our understanding of the mechanisms responsible for endogenous vascular repair has significantly been expanded. Until the late 1990s, vascular regeneration had exclusively been attributed to local processes, including proliferation and migration of mature vessel wall cells. In 1997, Asahara identified so-called endothelial progenitor cells (EPCs) which were capable to promote post-ischemic vascular regeneration in a direct manner (11). Meanwhile, it has become to attention that EPCs are represented by at least two distinct subpopulations. These differ in terms of origin and biological cell properties. Early EPCs (eEPCs) are most likely myelomonocytic in nature, acting by indirect mechanisms such as the release of humoral factors and vasomodulatory microvesicles (12, 13). According to newer concepts, eEPCs have meanwhile simply been defined as proangiogenic cells or PACs (14). Herein, we nevertheless continue to employ the term of eEPCs instead of PACs. Late EPCs (endothelial colony-forming cells—ECFCs) in contrast lack any hematopoietic properties and act within the vascular microenvironment by substituting damaged mature endothelial cells directly (15). Nevertheless, both populations have been shown as effective therapeutic tools in ischemic disease models, such as ischemic heart, cerebrovascular, and renal disease (12, 16–19). In addition, circulating eEPCs have been established as markers of impaired vascular function in several human disorders (9, 10, 20–22).

Several angiogenic mediators modulate the activity of EPCs. Of interest for this study were vascular endothelial growth factor (VEGF), angiopoietin 1, and CXCL16. Vascular endothelial growth factor and its receptor VEGFR are involved in angiogenesis under various conditions (23). In early phase DM for instance, higher VEGF expression has been documented in the fascia as compared to the muscle cells (24). Early disease stages of both DM and PM have also been associated with higher serum VEGF. The chemokine CXCL16 is expressed on endothelial cells, macrophages, dendritic cells, and cancer cells (25). The interaction of dextran sulfate sodium and CXCL16 activates the p38/Akt pathway, followed by stimulated angiogenesis (26). In addition, CXCL16 has been shown to promote VEGF production by modulating hypoxia-inducible factor-1 alpha (HIF-1a) (26). Angiopoietin 1 is essential for the maturation of blood vessels and regulates adhesion and migration of endothelial cells *via* Tie-2 receptor (27). Angiopoietin 1 has finally been shown to affect the activity of muscle cells *per se* (28).

Since DM is in part believed to be mediated by microvascular (endothelial) damage, we herein evaluated parameters of eEPC integrity in DM as compared to NM. The fundamental aim was to identify possible associations between impaired eEPC regeneration/blood cell numbers, (immuno)histological findings, and regulation of angiogenic mediators in myositis, potentially helpful to reduce the severity of microvascular and muscular damage.

## Materials and methods

### Patients

All patients with DM or NM were recruited from the Departments of Nephrology/Rheumatology, Neurology, or Dermatology of the University Medical Center of Göttingen (Germany). Recruitment was exclusively done by a specialist in Rheumatology/Neurology/Dermatology. The study was formally approved by the local ethics committee of the University Medical Center of Göttingen. All subjects signed written consent if they agreed to participate. All inflammatory myopathies were diagnosed according to the criteria established by Hoogendijk et al. Included patients were asked for general and myopathy-associated history and underwent physical examination and laboratory testing. For the latter, every individual supplied 10 ml of peripheral blood (lithium-heparin tubes). Autoimmune-related laboratory findings were extracted from the local databases of the University Hospital. The physical examination involved muscle strength analysis according to Janda (29). One half of the subjects underwent capillary microscopy. Assessment of the skin included the following parameters: Gottron's sign; lid edema; thoracic erythema; ungual hyperkeratosis. Cardiac involvement was defined as abnormal findings in the electrocardiogram, in cardiac ultrasound analysis, and laboratory findings as elevated Troponin T in the absence of other known causes. Lung disease was defined by a lower than normal diffusion capacity and/or by abnormal radiographic findings, and the involvement of liver and spleen was stated if laboratory testing revealed abnormal liver enzymes in the absence of other causes and/or if ultrasound analysis showed larger than normal organ dimensions.

The control group (Control non-m) consisted of healthy subjects and of 16 patients without the diagnosis DM or NM. These individuals provided blood for the analysis of circulating EPCs and of EPC colony formation. Since the ethics committee did not approve to obtain tissue samples from healthy donors, all control experiments in the immunofluorescence studies required the analysis of tissue samples, available in the department of pathology of the Göttingen University Hospital. The samples were derived from patients without inflammatory myopathy.

### Cytometric analysis and colony-forming assay

The respective procedures for cytometric quantification of CD133^+^/VEGFR-2^+^ cells have been published numerous times before (8–10, 30). In brief, mononuclear cells (MNCs) were isolated by density gradient centrifugation using Histopaque-1,077 solution (Sigma Diagnostics, St. Louis, MO) from ≈ 7.5 ml of peripheral blood (lithium-heparin tubes). Cells were incubated for 1 h on ice with one or more of the following antibodies: rabbit anti-CD133 (ab16518–Abcam, Cambridge, UK), mouse anti-human VEGFR2 (directly conjugated–FAB 3571F—R&D Systems, Minneapolis, MN, USA), followed by secondary incubation with PE-conjugated goat anti-rabbit Fab (VEGFR, 111-116-144—Jackson ImmunoResearch, Baltimore, PA, USA) for 30 min on ice. After incubation, cells were washed [PBS-BSA 1% (w/v)]. Data were acquired using a FACSCalibur cytometer (Becton Dickinson, Heidelberg, Germany) equipped with a 488 nm argon laser and a 635 nm red diode laser and analyzed using CellQuest software (Becton Dickinson, San Jose, CA, USA). The setup of FACSCalibur was performed according to the manufacturer's instructions using unstained and single-antibody stained cells. The specificity of staining was controlled by incubation with isotype-matched immunoglobulins. To quantify total peripheral endothelial cells, the numbers of VEGFR-2 positive cells, to quantify eEPCs, and the numbers of CD133/VEGFR-2 double-positive cells within the myelomonocytic cell population were counted.

The colony-forming assay was performed by using the EndoCult Liquid Medium Kit^®^ (STEMCELL Technologies, Vancouver, BC, Canada) per the manufacturer's protocol. MNCs were resuspended in complete EndoCult medium and seeded at 5 × 10^6^ cells/well on fibronectin-coated tissue culture plates (BD Biosciences, Rockville, MD, USA). After 48 h, wells were washed with media, and non-adherent cells were collected. Non-adherent cells were plated in their existing media at 10^6^ cells/well in 24-well fibronectin-coated tissue culture plates for 3 days. Only colonies with at least 20 cells, containing rounded cells in the middle and elongated cells at the periphery, were considered as CFU-EC colonies. The numbers of colonies (colonies/well) appearing after this period were counted. At least two members of the laboratory staff evaluated the numbers of colonies. They were blinded for the diagnosis and status of the investigated patients/controls. After counting the colonies, cells were acetone-fixated and frozen at −20°C.

### Fluorescence analyses

Muscle tissue samples were frozen at −80°C after formalin fixation and sucrose treatment, respectively. Freezing was performed in an OCT compound (SAKURA FINETEK, USA). Samples were cut using the Leica cryostat cm 3,000 (LEICA, Germany), and the thickness per section was 10–12 μm. After thawing, sections were washed three times with PBS, followed by blocking with PBS-goat serum 1% (w/v) for 10 min. In general, primary incubation was performed at 4°C overnight while secondary incubation was performed for 1 h at room temperature (RT). Antibodies were applied in PBS-goat serum 1% (w/v). Sections were repeatedly washed with PBS between primary and secondary incubation and afterward. Nuclei were counterstained with DAPI solution before covering the sections. All slides were kept at 4°C in the dark. The following antibodies were used for primary incubation: anti-Nestin (Abcam—ab 6,320, 1:1,000), anti-CD31 (Abcam—ab 28,364, 1:50), anti-MHC-class I (AbdSerotec—MCA485G, 1:200), and anti-CXCR6 (Dianova—CYT 74660, 1:500). The following antibodies were employed for secondary incubation: goat IgG anti-mouse IgG (Dianova-−115 585 003, 1:1,000), goat IgG anti-rat IgG (Dianova-−112 585 003), goat IgG anti-rabbit IgG (Dianova-−111 545 003), and goat IgG anti-rabbit IgG (Dianova-−111 585 003). Fluorescence images were acquired with the Carl Zeiss Axiovert S100 TV Inverted Microscope, equipped with the appropriate excitation and emission filters. Images were analyzed with the program Cell-D. Three images were analyzed per individual patients, respectively.

### Skeletal muscle pathology

Sections of frozen muscle samples (5 μm) were stained by hematoxylin/eosin. General muscle pathology was assessed by conventional light microscopy, including the quantification of inflammation, necrosis, atrophy, and other pathological criteria. Three images were analyzed per individual patients.

### Skeletal myotubes

For all primary skeletal myotube experiments, tissue samples were obtained from healthy donors. Individuals, scheduled for required knee surgery, were informed about the muscle biopsy, and muscle samples were collected for primary cell cultures. Some general information must be prepended: The isolation of myoblasts has been done *via* labeling with neural cell adhesion molecule (anti-CD56, mouse clone Eric-1; Neomarkers/Labvision), followed by magnetic bead–labeled secondary antibody for magnetic cell sorting. The purity grade was evaluated by immunocytochemical staining for the muscle marker desmin. In the experiments performed, the visual grade of purity was at least 90% for desmin-positive cells (myotubes). Other cell types are presumably fibroblasts. Staining for CD31 or CD45 has not been performed by standard, so it cannot be excluded that single endothelial cells were finally present.

Tissue preparation: The muscle tissue sample was minced and digested using trypsin. The fragments were seeded into a 25-cm^2^ flask in Dulbecco's modified Eagle's medium with pyruvate, high glucose, and L-glutamine (Gibco Invitrogen), supplemented with 10 % fetal calf serum (Cambrex Bioscience), penicillin, streptomycin (Gibco Invitrogen), and 0.5 % chick embryo extract (Accurate). After 2–3 weeks, myoblasts were labeled using a monoclonal antibody for CD56, a neural cell adhesion molecule (mouse clone Eric-1; Neomarkers/Labvision), followed by incubation with magnetic bead–labeled secondary antibodies to enable for magnetic cell sorting of positive cells. For further experiments, myoblasts were seeded in 24-well plates (Nunc) at 80 % confluence. Well-differentiated myotubes, as revealed by immunocytochemical staining specific for the muscle marker desmin, were either kept as unstimulated controls in X-Vivo 15 medium (Cambrex Bioscience) or exposed to the serum of patients with dermatomyositis (DM) and necrotizing myopathy (NM) at different concentrations, ranging from 1:45 to 1:5 in medium X-vivo 15 (Cambrex Bio Science) for 24 h at 37°C using a humid incubator.

### RNA extraction and RT-PCR

Extraction of total RNA was carried out following the supplier's instructions (RNeasy Kit, Qiagen). For cDNA synthesis, the SuperScript II reverse transcriptase kit (Invitrogen) was employed following the supplier's instructions. The resulting cDNA was stored at −20°C. Quantitative (real-time) PCR was performed on a 7,500 real-time PCR system using 6-carboxyfluorescein (FAM)-labeled specific primer/probe pairs for glyceraldehyde-3-phosphate dehydrogenase (GAPDH, Hs99999905_m1), CD31 (Hs01065279_m1), nestin (Hs04187831), IL-1β (Hs00174097_m1), and IL-6 (Hs00174131_m1) (from Applied Biosystems). Target mRNA expression was quantified using the 2^ΔΔc(t)^ method in relation to the expression of the housekeeping gene GAPDH.

### ELISA studies

Commercially available ELISA tests were used for the assessment of angiopoietin 1, vascular endothelial growth factor (VEGF), and CXCL16 (all from R&D systems, MN, USA). Tests were performed according to the manufacturer's protocol.

### Biochemical and hematological tests

Biochemical and hematological tests were performed in the Central Laboratories of the University medical center of Göttingen, according to the institutional guidelines.

### Statistical analysis

For statistical analyses, we employed the program “R.” For comparing more than two groups, the Kruskal–Wallis test (analysis of variance—ANOVA) was applied; two individual groups were compared with the Wilcoxon rank sum test. The results were depicted by either dot or box plots, depending on the number of measurements. The median was marked in every series. Significant differences were assumed if *p*-values were below 0.05.

## Results

### Patients and controls

The following groups were included: dermatomyositis—DM, necrotizing myopathy—NM, and controls [herein: Control non-m (non-myopathy)]. The control group (Control non-m) consisted of 24 healthy subjects and of 16 patients with the following diagnoses: inclusion body myositis—*n* = 5, chronic idiopathic demyelinating polyneuropathy—*n* = 5, myotonic dystrophy—*n* = 5, and multifocal polyneuropathy—*n* = 1. Six subjects that did neither suffer from DM or NM but received muscle biopsy for diagnostic purposes. Two individuals with muscle biopsy suffered from chronic idiopathic demyelinating polyneuropathy (CIDP), and one individual was diagnosed with multifocal polyneuropathy. Therefore, two individuals (CIDP) from the Control non-m group received permanent prednisolone therapy at the time of biopsy, and the respective dose was <7.5 mg daily, respectively. The following numbers of subjects were included in the study: controls 40; DM 17; and NM 7. The mean age (years ± SD) in the respective groups was controls 44 ±12; DM 56 ±14; and NM 47 ±16. The male:female ratio was controls 10:30; DM 6:11; and NM 3:4. Muscle biopsy was performed in controls 12,5; DM 59; and NM 86%. Immunosuppressive therapy at the time of inclusion into the study was performed in controls 9; DM 77; and NM 71%. All patients underwent laboratory analysis for C-reactive protein, liver enzymes (AST and ALT) and muscle enzymes (creatine kinase), blood count, and myositis autoantibodies. In addition, patients were evaluated for malignant diseases. Cancer-associated myositis was diagnosed in DM 29% and NM 0%. Table 1 summarizes the clinical characteristics of all control subjects and patients.

**Table 1:** Detailed patients' characteristics.

	**DM**	**NM**	**Controls**
number of patients / subjects	17	7	40
mean age at diagnosis (years ± SD)	56 ± 14	47 ± 16	44 ± 12
M : F	6 : 11	3 : 4	10 : 30
muscle biopsy	59%	86%	12.5%
mean duration of the disease (years)	5.6	2.6	0
ANA (positiv)	59%	36%	not measured
daily prednisolone dose	88%	86%	7.5%
<7.5 mg	64%	43%	7.5%
≥7.5 mg	24%	43%	0%
immunosuppressive therapy	77%	71%	7.5%
smoking	29%	29%	unknown
neoplasia	29%	0%	0%
**autoantibody profile**			
Mi-2	29%	0%	0%
other, non-Mi-2 autoantibodies	24%	67%	0%
HMGCR	0%	17%	0%
SRP	0%	33%	0%
**laboratory findings**			
CK (U/l) (mean ± SD)	3,696 ± 4,719	6,819 ± 4,796	222 ± 83
hypothyroidism	18%	29%	0%
AST (mean U/L ± SD)	164 ± 180	193 ± 200	unknown
ALT (mean U/L ± SD)	147 ± 170	108 ± 105	unknown
LDH (mean U/L ± SD)	605 ± 397	992 ± 651	unknown
CRP (mean mg/L ± SD)	13 ± 28	12.4 ± 8	unknown
leukocytes (× 10^3^/ul) (mean ± SD)	8 ± 4	12 ± 6	unknown
**organ involvement**			
**muscle strength according to Janda**			not evaluated
axial (mean ± SD)	4.8 ± 0.4	4.8 ± 0.4	
proximal (mean ± SD)	4.5 ± 0.5	4.6 ± 0.6	
distal (mean ± SD)	4.7 ± 0.7	4.8 ± 0.4	
**skin involvement**	94%	0%	0%
Gottron's sign	35%	0%	0%
lid edema	41%	0%	0%
rash (décolleté)	82%	0%	0%
ungual hyperceratosis	35%	0%	0%
dysphagia	47%	0%	0%
dyspnea	24%	14%	0%
morning stiffness	41%	57%	2.5%
**other inner organs**	53%	29%	0%
heart	29,4%	14%	0%
lungs	24%	14%	0%
liver	6%	29%	0%
spleen	12%	14%	0%

### eEPC regeneration is impaired in inflammatory myopathies

**Figure 1:**
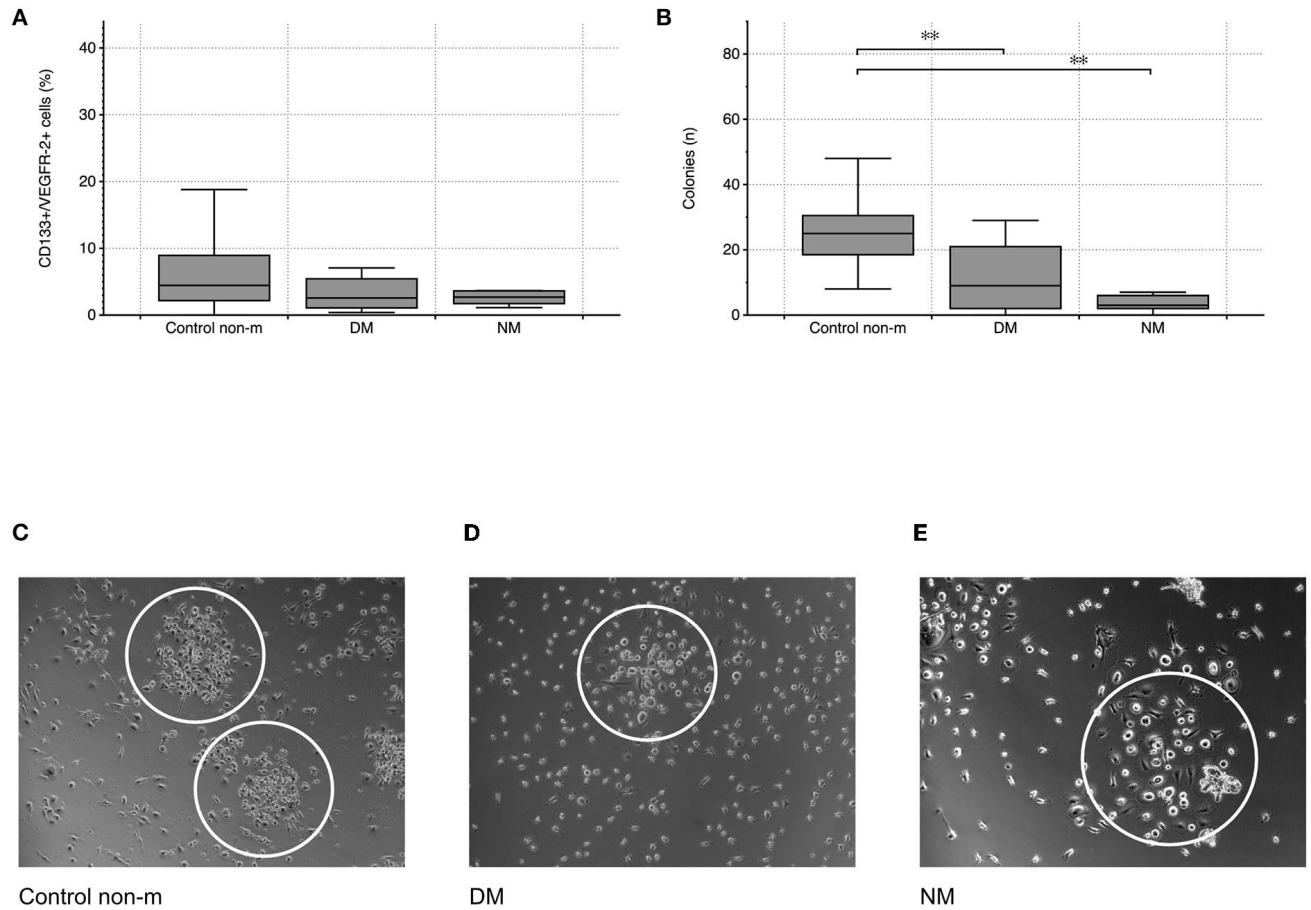
Cytometric quantification of peripheral circulating eEPCs (CD133+/VEGFR-2+ cells) indicated lower percentages in the DM group without reaching the level of significance **(A)**. Blood-derived eEPC colonies were significantly lower in the two disease groups **(B)**. Images **(C–E)** depict representative patterns of colony formation in controls and in the disease groups (DM and NM). The white ovals surround individual colonies. The following numbers of samples were analyzed per group: Control non-m: n = 40; DM: n = 15; NM: n = 6 (Control non-m: non-myopathic control; magnifications: ×20 in Control non-m and in DM; ×40 in NM;^*^, p-value < 0.05; ^**^, p-value <0.01).

Cytometric analysis did not reveal any significant differences in CD133^+^/VEGFR-2^+^ cells between controls and patients with either DM or NM, respectively (peripheral circulating eEPCs—Figure 1). In contrast to circulating eEPCs, colony numbers significantly differed between controls and the disease groups. The latter showed lower colony formation capacity, indicating impaired regenerative activity of blood-derived eEPC: *p*-value of controls vs. DM 0.004; *p*-value of controls vs. NM 0.001 (colony-forming units (CFU)—Figure 1).

### DM is associated with intramuscular capillary rarefaction

**Figure 2:**
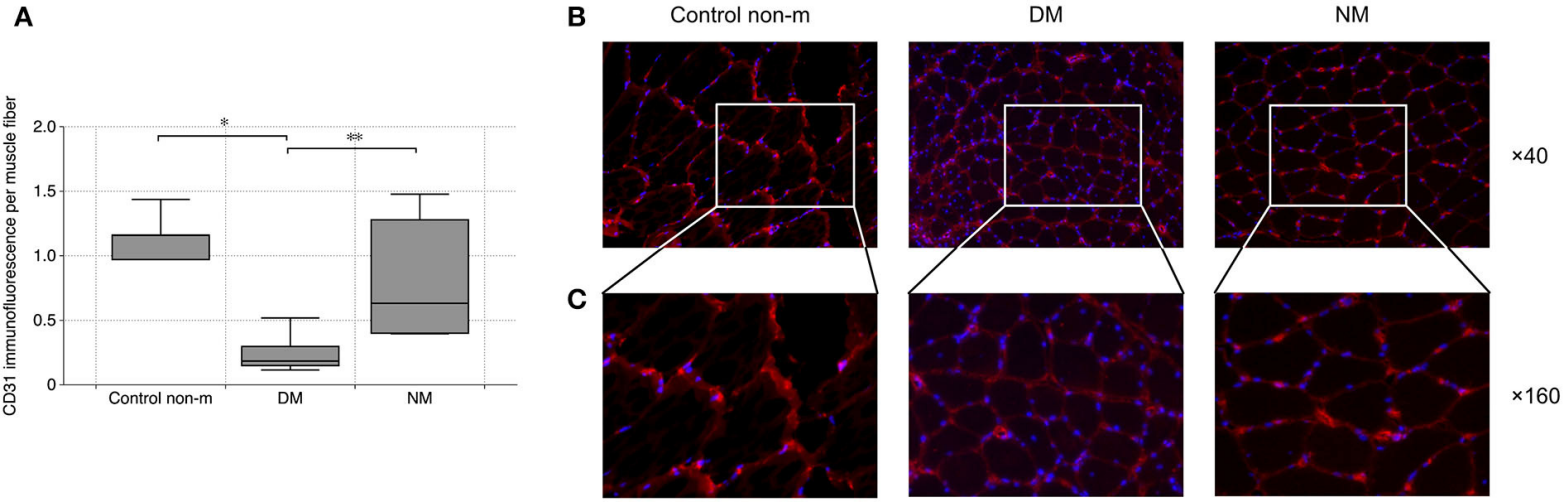
Analysis of vascular density. Vascular density was evaluated by quantification of CD31 immunofluorescence per muscle fiber. The analysis **(A)** showed a significantly lower vascular density in DM as compared to the Control non-m group. In addition, DM patients displayed lower vascular density in comparison with NM. Fluorescence images **(B)** show representative sections after CD31 staining (CD31 (red), magnification ×40). Panel **C** shows magnified areas from each group (×160), and they illustrate the loss of CD31 in DM as compared to Control non-m and to NM more in detail. The following numbers of samples were analyzed per group: Control non-m: n = 6; DM: n = 16; NM: n = 6 (Control non-m: non-myopathic control; magnification in all images ×40; ^*^, p-values <0.05; ^**^, p-value <0.01).

To evaluate vascular density within the skeletal muscle, dot-like CD31^+^ signals were related to the number of individual muscle fibers and to the overall staining area (per muscle fiber). DM patients displayed significantly diminished vascular density, compared to controls. DM patients showed lower vascular density than NM (Figure 2).

### DM and NM are characterized by endothelial cell activation

**Figure 3:**
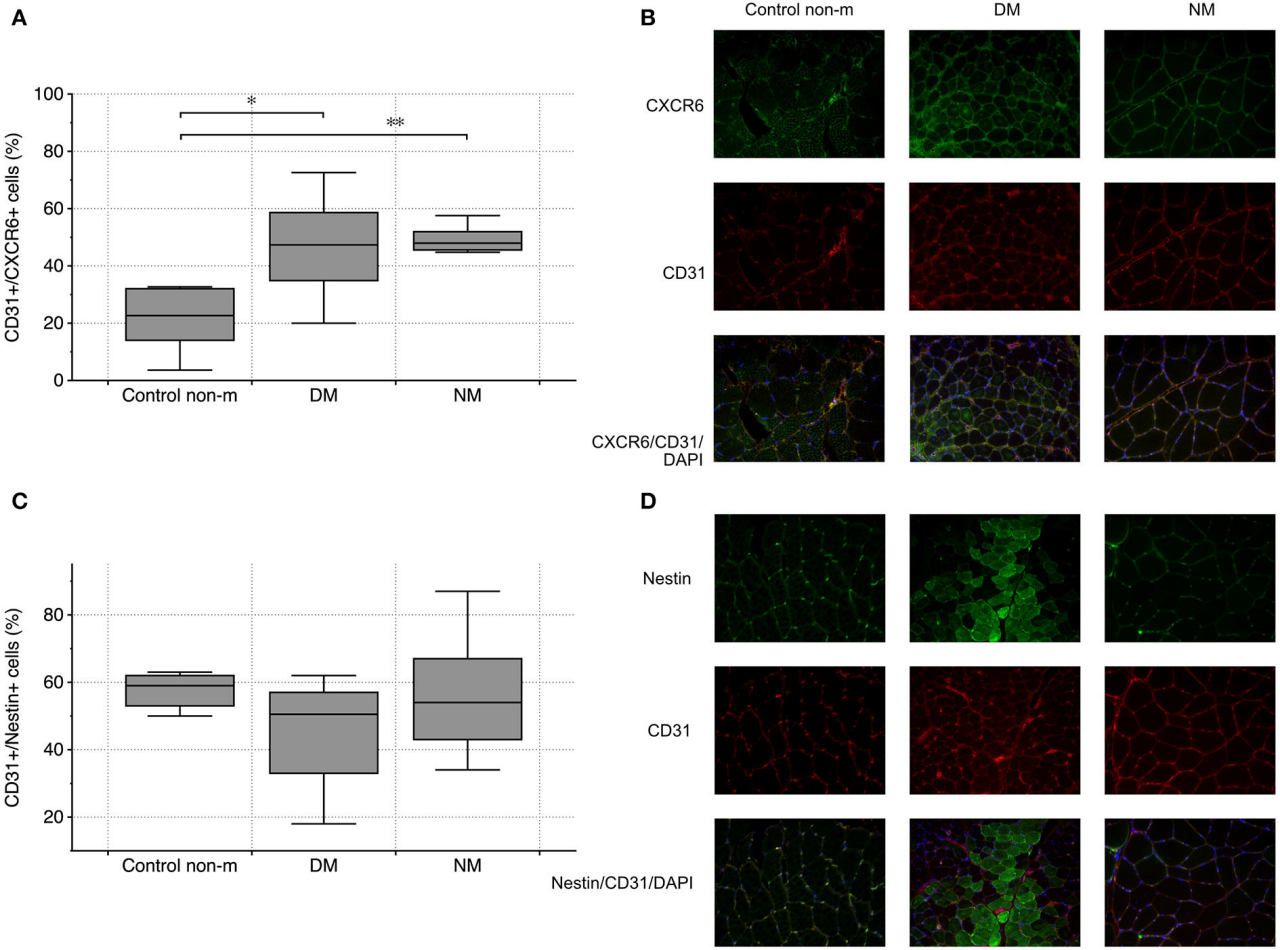
Vascular CXCR6 expression and endothelial nestin abundance in controls (Control non-m) and in all disease groups. Endothelial (CD31+) cells showed more intense staining patterns for CXCR6 in DM and NM than in controls **(A)**. The image panel [right-sided—**(B)]** shows representative images from the respective groups after staining for CXCR6 (green), CD31 (red), and after merging including the nuclei (blue). Endothelial nestin did not differ between any of the groups **(C)**. The fluorescence images on the right depict staining patterns of nestin and CD31 and of both markers combined (nestin (green), CD31 (red), and after merging, nuclei (blue)) **(D)**. The following numbers of samples were analyzed per group: Control non-m: n = 6; DM: n = 10; NM: n = 6 (Control non-m: non-myopathic control; magnification ×40; ^*^ and ^**^, p-values <0.05).

Endothelial CXCR6 expression most likely reflects a state of cell activation, intended to recruit blood-delivered eEPCs to areas of vascular damage and repair. In both disease groups (DM and NM), endothelial CXCR6 expression was significantly higher than in control subjects (Figures 3A,B). Muscle fibers were evaluated for CXCR6 expression as well. Increased or reduced CXCR6 positivity was not detected in any of the groups (not shown). To evaluate whether differences of muscular CXCR6 expression possibly result from T-cell infiltration, sections were additionally stained for CD4 and CD8, respectively. Differences in CXCR6^+^/CD4^+^ or CXCR6^+^/CD8^+^ cells were not significant between any of the groups (not shown).

Nestin has been established as marker of mesenchymal cell regeneration. A number of studies showed the protein to be expressed in differentiating cells and in cells that recover from acute functional impairment/structural damage (e.g., ischemia) (31, 32). Muscle and endothelial cells do not express nestin under normal circumstances (33). Our analysis did not show differences of CD31^+^/nestin^+^ cells between the respective groups (Figures 3C,D).

### Serum levels of (pro)angiogenic mediators remain unaffected in DM and NM

**Figure 4:**
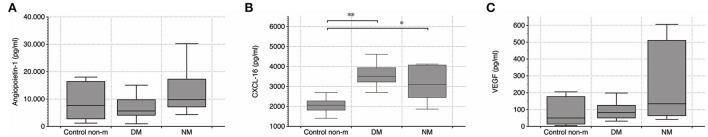
**(A–C)** Quantification of serum Ang-1, CXCL16, and VEGF by ELISA. Systemic VEGF level did not differ between any of the groups, but CXCL16 was elevated in DM and NM, respectively. Further differences were not detected. The following numbers of samples were analyzed per group: Control non-m: n = 11; DM: n = 14; NM: n = 6 (Control non-m: non-myopathic control; ^*^ and ^**^, p-values <0.05).

Serum levels of vasomodulatory angiopoietin 1 (Ang-1) and vascular endothelial growth factor (VEGF) were evaluated. Additional analyses intended to quantify serum CXCL16, the ligand of CXCR6. Both VEGF and Ang-1 did not differ between any of the respective groups at all. CXCL16 was significantly elevated in the two disease groups (Figure 4), which supports the notion that CXCR6 signaling is crucial in myositis.

### Myotubes display endothelial and pro-inflammatory properties under pro-inflammatory conditions

**Figure 5:**
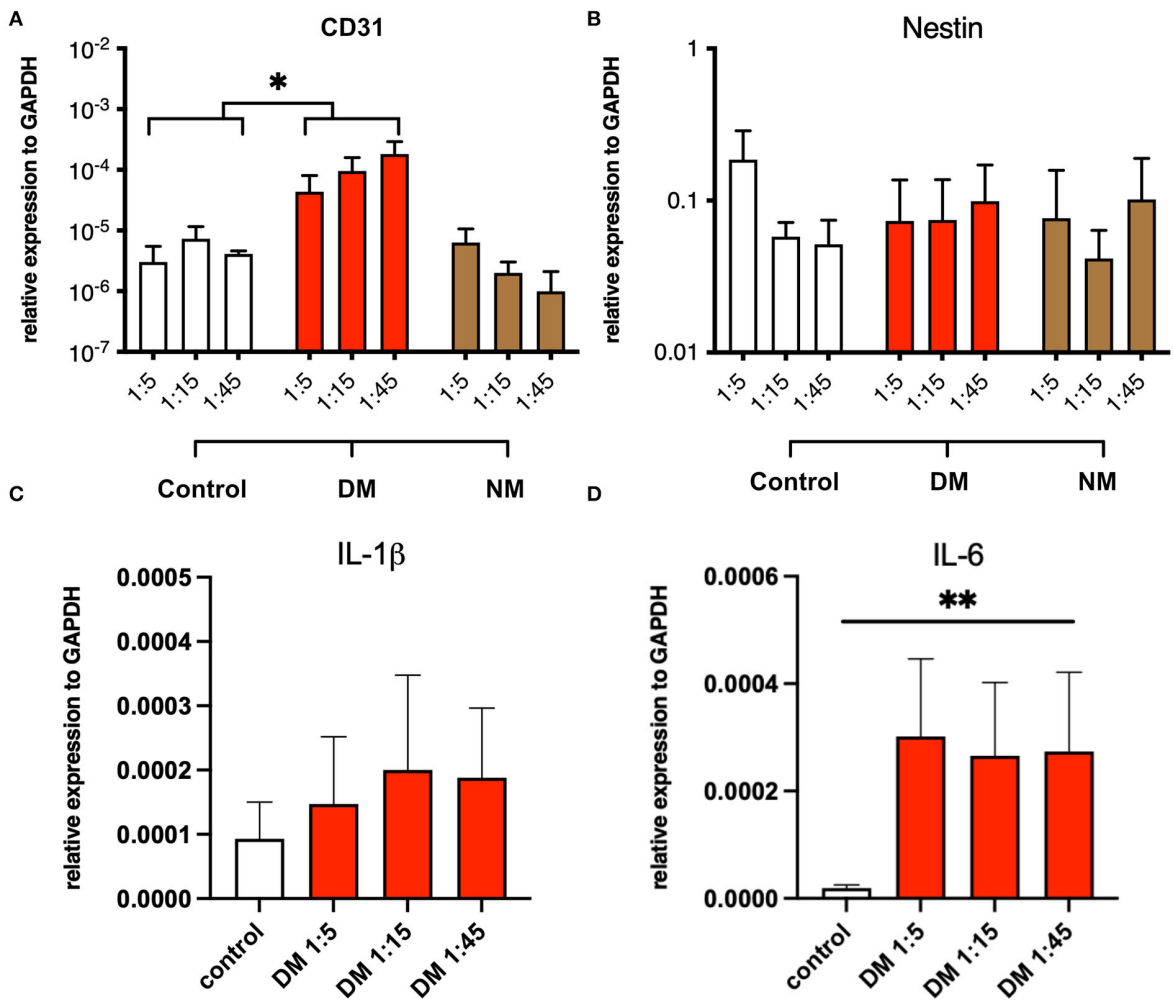
Serum stimulation of cultured myotubes from DM and NM patients. **(A)** PECAM-1 mRNA expression was significantly enhanced upon stimulation with serum derived from DM patients. Since control samples showed low CD31 abundances also, residual presence of CD31+ cells must be considered (^*^, p-value <0.05). **(B)** Nestin mRNA expression levels did not change in a significant manner upon serum treatment. **(C)** A trend toward higher IL-1b mRNA expression was noticed without reaching statistical significance. **(D)** IL-6 mRNA expression was significantly upregulated (^**^, p-value <0.01).

The modulation of pro-inflammatory mechanisms by serum, derived from DM and NM patients, was assessed in myotube cultures from non-myopathic donors. Myotubes were serum-incubated at different concentrations and analyzed for mRNA expression of the following proteins: CD31, nestin, IL-1β, and IL-6. While the abundances of nestin and IL-1β mRNA did not differ between the groups, CD31 and IL-6 mRNA were significantly increased upon exposure to serum from DM patients but not after NM serum exposure stimulation. The pro-inflammatory properties of DM-derived serum persisted after serial dilution (Figure 5).

## Discussion

Inflammatory myopathies are represented by diseases with heterogeneous etiology / pathogenesis, each characterized by distinct (immuno)pathological findings. Abnormal vascular pathology has been identified in DM (5). Nevertheless, complement depositions of C5b-9 MAC were evident in skeletal muscle fibers from DM and NM subjects (5). The endothelial progenitor cell system has been proven to mediate vascular repair under diverse conditions. Responsible mechanisms include direct and indirect effects, the latter being mediated by the release of vasoprotective proteins and proangiogenic microvesicles in a paracrine manner (13). The discussion about the true nature of EPCs continues (34). According to newer concepts, ECFCs (endothelial colony-forming cells) must be distinguished from eEPCs (early endothelial progenitor cells); for the latter, the term PACs (proangiogenic cells) has been proposed instead of eEPCs (14). In the current study, we evaluated eEPCs/PACs and continued to employ the initial term (12, 13). While our study did not reveal significant differences in circulating eEPCs as compared to controls, eEPC regeneration, as reflected by the number of colonies in culture, was significantly reduced in both myositis subsets. Vascular density was diminished in DM, the overall tissue and endothelial contents of proangiogenic CXCR6 were elevated in DM and NM, whereas endothelial nestin abundances remained within the range of healthy controls. Serum levels of CXCL16, the ligand of CXCR6, were higher in both disorders, indicating the activation of proangiogenic repair not exclusively in DM. Thus, particularly in DM, with induction of intramuscular endothelial damage (vascular rarefaction) and activation (increased endothelial CXCR6), the endothelial repair machinery is activated but severely affected (CXCL16 elevation, lower eEPC colony formation, constant serum VEGF and Ang-1, and constant endothelial nestin). Interestingly, DM-derived serum induced endothelial and pro-inflammatory properties in cultured myotubes. Any potential relevance of these findings with regard to the pathogenesis of myositis-associated microvascular damage needs to be further elucidated.

Ekholm and colleagues found reduced percentages of circulating eEPCs in myositis (DM and PM) (35). The cellular differentiation capacity toward an endothelial phenotype was significantly affected (35). The patients displayed higher type I interferon (IFN I) serum levels which were in line with histological observations made by Greenberg et al. (36). It needs to be mentioned that Ekholm and colleagues reported eEPC-neutralizing effects of both anti-IFN I and anti-IL-18 (35). As pointed out by Kahlenberg et al., the type I interferon axis exhibits detrimental effects on the differentiation and growth of eEPCs (37). Consequently, proangiogenic IL-1β was shown to be reduced (38), and IL-18, a cytokine with inhibitory effects on eEPCs, was enhanced (37), (39). Ekholm et al. already analyzed neutralizing effects of antibodies against type I IFN receptor and IL-18 in patients with DM and PM and showed reverse effects on eEPCs if both antibodies acted in combination (35).

The pathology of DM includes C5b-9 MAC (membrane attack complex)-mediated vascular damage (40). Vascular damage is typically reflected by intramuscular capillary reduction (vascular rarefaction—VR) (41). One may hypothesize that impaired regeneration of blood-derived eEPCs and VR is linked to each other in a mechanistic manner, although our data do not offer any proof for such a concept. The current literature discusses alternative mechanisms that may account for the pathology in DM. Thus, a certain type of vascular narrowing of the perimysial arcade arteries has been reported by Gitiaux et al. (42). The respective perivascular areas were infiltrated by pro-inflammatory cells. Complement activation and microvascular damage have also been reported to occur in NM (5). Microvasculopathy in this disease is characterized by so-called “pipestem capillaries” (43). However, in contrast to DM, we did not detect lower vascular density in NM. Such observation does not exclude significant VR to occur during *later* stages of the disease. It needs to be mentioned that the mean duration of the disease in NM patients was 2.5 years.

Nestin, a constituent of the cytoskeleton, is increasingly expressed in certain types of (progenitor) cells that proliferate/regenerate (44). In this context, the protein has been detected in endothelial cells of the brain and in other organs, such as the post-ischemic kidney (31, 32, 45). Thus, nestin may be regarded as marker of (mesenchymal) regeneration. Our analyses did not show significant differences of CD31^+^/nestin^+^ cells between the respective patient groups. Nevertheless, DM subjects tended to lower endothelial nestin abundances. It remains speculative whether this particular observation reflects impaired endothelial regeneration as contributing factor of microvasculopathy. Nestin is expressed in embryonic but not in adult muscle cells (33). Nevertheless, the protein has been shown to re-appear in muscle cells several h after injury.

Isozaki (46) discussed the possibility that CXCR6, besides mediating lymphocyte recruitment and adhesion, is also capable to activate mesenchymal stem cells and to participate in vasculogenesis. CXCL16, a transmembrane protein with adhesion functionality, is the only known CXCR6 ligand. It is expressed on macrophages, dendritic cells, smooth muscle, and endothelial cells (47). CXCL16 has been shown to activate mature endothelial cells (48). We therefore analyzed serum CXCL16 levels and found significant increases in both disease groups with the highest elevation in DM. It needs to be mentioned that comparable findings were reported in patients with systemic sclerosis (SSc). A study by Yanaba and colleagues (49) revealed a correlation between serum CXCL16 and the extent of skin involvement in SSc. Despite these findings, the overall capillary density was decreased in DM. In contrast, well-known proangiogenic mediators such as VEGF and angiopoietin 1 did not show any upregulation. The vascular potential of CXCL16 therefore needs to be discussed. It remains uncertain whether VEGF/angiopoietin 1 activation is either inadequate, or dissipated or disrupted.

In summary, our study revealed the loss of intramuscular microvessels in DM, accompanied by endothelial activation, which was similarly present in NM. Vascular regeneration was impaired in DM and NM. Yet, exogenously induced inflammation, in conjunction with an endothelial trigger signal, was induced by serum from DM but not NM. Collectively, our findings support a specific role for myositis-associated vascular damage in the pathogenesis of DM.

## Data availability statement

All data are available upon request to spatschan@gmail.com.

## Ethics statement

The studies involving human participants were reviewed and approved by Ethics committee of the University Medical Center of Göttingen. The patients/participants provided their written informed consent to participate in this study.

## Author contributions

DL collected all data. JS participated in designing the study and provided financial support. KK performed all myotube experiments. BL performed statistical analyzes. AW assessed muscle sections. CS helped in patient recruitment. KS performed ELISA experiments. GM supported the study financially. DP wrote the manuscript. SP designed the study, recruited patients, and assisted in writing the manuscript. All authors contributed to the article and approved the submitted version.

## Conflict of interest

The authors declare that the research was conducted in the absence of any commercial or financial relationships that could be construed as a potential conflict of interest.

## Publisher's note

All claims expressed in this article are solely those of the authors and do not necessarily represent those of their affiliated organizations, or those of the publisher, the editors and the reviewers. Any product that may be evaluated in this article, or claim that may be made by its manufacturer, is not guaranteed or endorsed by the publisher.
